# Analysis of mesothelioma cases and National Cancer Registry data to assess asbestos exposure in India

**DOI:** 10.5588/pha.24.0003

**Published:** 2024-03-01

**Authors:** R. Singh, A. L. Frank

**Affiliations:** Department of Environmental and Occupational Health, Dornsife School of Public Health, Drexel University, Philadelphia, PA, USA

**Keywords:** cancer, mesothelioma, cancer record-keeping, asbestos

## Abstract

**SETTING:**

Asbestos exposure can cause mesothelioma, a form of cancer which should be recorded by cancer registries. However, such registries currently cover only a small fraction (16%) of the population in India. Because India still uses asbestos, it is important to understand its health impact, especially the number of mesothelioma cases.

**OBJECTIVE:**

To assess the number of mesothelioma cases in India and compare these to the number reported to the National Cancer Registry.

**DESIGN:**

We used the Right to Information Act 2005 to gather data for 83 hospitals across India from 2012 to 2022-2023.

**RESULTS:**

From a total of 83 hospitals, there were 2,213 cases of mesothelioma from 2012 onwards. During the 2012–2016 period, the number of reported cases in the Cancer Registry was 54, whereas 1,126 cases were reported by these hospitals for this period. Only 21 (25%) of the hospitals assessed in this study were part of the population-based national cancer registry programme. Overall, cases of mesothelioma occur far more frequently than are reported in cancer registries.

**CONCLUSION:**

National record-keeping is inadequate and the system needs to be expanded and improved across all of India. This will provide more effective reporting and help to highlight the risk of exposure to asbestos.

## Abstract (fr)

**CONTEXTE:**

L'exposition à l'amiante peut provoquer un mésothéliome, une forme de cancer qui devrait être répertoriée dans les registres du cancer. Cependant, ces registres ne couvrent actuellement qu'une petite fraction (16%) de la population indienne. L'Inde utilisant encore l'amiante, il est important de comprendre son impact sur la santé, en particulier le nombre de cas de mésothéliome.

**OBJECTIF:**

Évaluer le nombre de cas de mésothéliome en Inde et le comparer au nombre de cas déclarés au Registre National du Cancer.

**CONCEPTION:**

Nous avons utilisé la loi de 2005 sur le Droit à l'information pour recueillir les données de 83 hôpitaux indiens entre 2012 et 2022–2023.

**RÉSULTATS:**

Sur un total de 83 hôpitaux, 2 213 cas de mésothéliome ont été recensés à partir de 2012. Au cours de la période 2012–2016, le nombre de cas signalés dans le Registre du Cancer était de 54, alors que 1 126 cas ont été signalés par ces hôpitaux pour cette période. Seuls 21 (25%) des hôpitaux évalués dans cette étude faisaient partie du programme national de registre du cancer basé sur la population. Dans l'ensemble, les cas de mésothéliome sont beaucoup plus fréquents que ceux signalés dans les registres du cancer.

**CONCLUSION:**

La tenue des registres nationaux est inadéquate et le système doit être étendu et amélioré dans toute l'Inde. Cela permettra d'améliorer l'efficacité des rapports et de mettre en évidence le risque d'exposition à l'amiante.

Mesothelioma is a form of cancer that mainly affects the lining of the lungs and is typically associated with exposure to asbestos.^1–4^ It may occur in the pleura or peritoneum, although the latter is less frequent.^5^ An estimate of the deaths from mesothelioma (based on incomplete national mortality data), indicates that there are approximately 38,400 mesothelioma-related global deaths per year.^6^ The academic literature indicates that malignant pleural mesothelioma ‘is a rare tumour in the Western world and still rarer in India’.^7^ In comparison with other forms of cancer, there are a relatively small number of cases, and mesothelioma has been classified as being rare in India.^8^ In a case report from 2019, an Indian physician discusses a 42-year-old man who died due to pleural mesothelioma with evidence of prior asbestos exposure, but stated that ‘there is no report from India of mesothelioma related to asbestos’.^9^ This quote was based on the statistics and health information systems of the 2018 WHO Mortality Database. Although this may be due to the small number of mesothelioma cases compared to other cancers, it is unlikely there is a complete absence of cases in India. Indeed, a retrospective study at the Department of Onco-Pathology at the Gujarat Cancer Research Institute from 2015 to 2019, reported 128 cases of malignant mesothelioma.^10^ This lack of consistency can be attributed to issues with cancer record-keeping in India. In addition to cancer being a non-notifiable disease, it has been reported that there is a ‘very poor, almost non-existent, system to record death and disease’.^11^ A national cancer registry programme was first launched in December 1981, under the leadership of the Indian Council of Medical Research. However, currently only 16% of the population is under the National Cancer Registry (although a figure of 10% has also been reported).^12,13^ The Cancer Registry Programme is run by the National Centre for Disease Informatics Research (NCDIR), Bengaluru, India, and has two types of registry systems:^14^ the hospital-based cancer registries (HBCRs); and the population-based cancer registries (PBCRs). A committee set up by the Indian Parliament on cancer-related matters ‘strongly’ believed that ‘there is an urgent need to have more rural PBCRs to get realistic information about the incidence and type of cancers across the country.’ HBCRs have been set up in tertiary care centres with large capacity and are usually dedicated centres for cancer. Some of these centres also take up the record-keeping work for hospitals in the same immediate geography, and these also operate PBCRs. In July 2022, the NCDIR stated that there are 38 PBCRs covering both urban and rural areas, but the last published report is for data up to 2016. As per the NCDIR, the data from 2016 onwards is still being processed and is not available. Although the number of PBCRs in 2016 was 36, the report included data from only 28, as only these PBCRs provided data which were ‘complete and met the desired quality’.^15,16^ It is important to note that PBCRs collect data from multiple sources, including various clinics in multiple hospitals of various specialties, palliative care units, diagnostic centres, as well as mortality data from death record centres.^16^ Furthermore, the data are not available for policy, research or epidemiological purposes unless published as a report in the public domain.^17^

Because post-2016 data were not available, and multiple hospitals were not under NCDIR coverage, we used the Right to Information Act 2005 (hereinafter, referred to as the RTI Act) to find data from various hospitals, and compare these to data from the National Cancer Registry. This has allowed us to better understand the number of mesothelioma cases and ascertain realistic figures for asbestos exposure in India.

## METHODS

We used the RTI Act to collect data from 83 hospitals across India.^18,19^ Hospitals were asked to provide the number of diagnosed cases per year from 2012 to 2022 (some were extended to 2023). The applications were specifically made for data on mesothelioma, which is classified under the International Classification of Diseases (ICD-10) as C45.^20^ The period of collection of information was the middle to late 2023. We received replies from hospitals that were public in nature, either under the State government or the Central government, or were substantially funded by these. This is because the RTI Act is valid only for ‘public authorities’.^18^ Some replies were incomplete and further information was required – we appealed under the act and the final data set received from all the hospitals were collated. Not all of the 83 hospitals were under the PBCR, which afforded us access to data, which has not been previously reported. The collected data were assessed to provide insights into the real number of mesothelioma cases in India. This provides fresh insight into this disease, which is almost always associated with people having had previous exposure to asbestos, either in occupational or non-occupational settings.

The data collected was also compared with data available in the NCDIR report from 2012 to 2016 (the time period for which published NCDIR data were available).^16^ The comparison was with respect to the number of cases, as well as the hospitals that were covered by the NCDIR database. There are a total of 525 centres listed for treatments related to cancer under the radiation license by the Atomic Regulatory Board in India.^21^ For calculating the number of hospitals (government or private) providing cancer treatment, this is considered the total size. According to this, 83 hospitals represent a sample size with a confidence level of 95% ±10% margin of error. An advantage of the RTI Act is that data provided are by default non-personal that have been released into the public domain. This study is therefore exempt from ethics review, and the data are available on request to the authors. An earlier version of this manuscript was submitted to the preprint server *medRxiv* on 19 December 2023.

## RESULTS

This study set out to establish a realistic figure for the number of mesothelioma cases in India. From the 83 hospitals under study, the total number of mesothelioma cases from 2012 to 2022 (and in some cases until 2023), was 2,213 cases (Table). The list of hospitals that were included in the study are listed in Supplementary Table S1. A comparison was also made between the cases recorded in this study and those registered by the Indian National Cancer Registry Programme (by NCDIR) between 2012 and 2016.^16^ Although the NCDIR recorded 54 cases, our study recorded 1,126 cases for the same period. The reasons for the difference in cases, along with the limitations in the NCDIR report, are discussed below. It is important to note that of the 83 hospitals included in our study, only 10 have a HBCR under the NCDIR programme. Apart from this, only 21 (25%) of the 83 were part of a NCDIR PBCR programme until the time of data release by NCDIR.^16^ This means that 62 (∼75%) of the hospitals are not part of the formal cancer record-keeping mechanism under the PBCR, and 73 (∼88%) of the hospitals do not have a HBCR under the programme. One state, Tamil Nadu, had a state-based cancer registry, which included 17 hospitals.^22^ For this State, the hospitals included in the registry have not been separately added, but as part of the total registry numbers. Data for other hospitals in Tamil Nadu that are not part of the state registry, have been added directly to this study.

**Table.** Year-wise count of mesothelioma cases from 83 hospitals.

	Mesothelioma C45 cases*
Year	*n*
2012	222
2013	263
2014	325
2015	147
2016	185
2017	170
2018	237
2019	147
2020	144
2021	158
2022	170
2023^†^	47

* As several hospitals provided aggregated data for multiple years, we used average values for those years, which may not accurately represent the data (see Supplementary Table S1 for a comprehensive listing for each hospital).

† Data for 2023 is not for the full year as the data were collected before the end of 2023.

## DISCUSSION

As has previously been noted, the Cancer Registry Programme in India is in a poor state.^23^ Its coverage is low and there are a large number of hospitals that are not included – this is not appropriate for a programme that was first started in 1981. Because each State is responsible for health in India, it is up to each individual State to decide whether to participate in a national programme. However, by making cancer a notifiable disease, as was recommended by the Parliamentary Standing Committee, participation could have been made mandatory, rather than on a voluntary basis^13^ It is pertinent to note that many States have made cancer notifiable. As an example, Tamil Nadu has a fairly robust cancer registry system. However, the Department of Health Research has declined to make cancer notifiable at the national level on the grounds that cancer is a non-infectious, non-communicable disease that does not have community spread and ‘in the present circumstances, it may not be declared as notifiable disease.’^12^ Other States (including Andhra Pradesh, Chhattisgarh, Goa, Haryana, Himachal Pradesh, Jharkhand, Odisha and Rajasthan) and Union Territories (including Andaman Nicobar Islands, Chandigarh, Dadra and Nagar Haveli, Daman and Diu, Ladakh, Puducherry and Lakshadweep) do not have PBCR.^15^ However, major hospitals in some States without a PBCR are listed in the PBCR of an adjoining state. It must also be noted that state-level cancer data, even if fully calculated, would not provide the complete picture, as people may move from one State to another for treatment. An example of this is Gujarat, which has the Gujarat Cancer and Research Institute (GCRI). From 2012 to July 2023, the total number of mesothelioma cases recorded at the GCRI was 303, 99 (∼32.67%) of which were from the adjoining state of Rajasthan and 23 (7.59%) from Madhya Pradesh. There were an additional 6 (∼1.99%) cases from other States, and the total case load from Gujarat was 175 (∼57.75%). This shows that the catchment area of certain established hospitals transcend state boundaries, and the records for these states may not be linked to the causative factors in those states. Furthermore, the data in this study were exclusively from government or public hospitals (as the RTI Act only applies to ‘public authorities’) and did not cover several private hospitals. It is highly likely that more cases would have been reported if private hospitals with cancer facilities had been included. One exception to this is the Tamil Nadu Cancer Registry, which includes data for private hospitals, and we therefore used the combined registry data, instead of an individual hospital-wise breakdown. Due to its limited coverage, the cancer registry does not provide a complete picture of the scene in India. This underreporting has serious consequences, as it is submitted to the International Cancer Registry and becomes the basis of global policy decisions.^24^

It is important to note that asbestos exposure can occur through occupational exposure (e.g., in a mine, an asbestos factory, a ship dismantling unit or brake lining unit) or non-occupational exposure (e.g., working in a building with asbestos, or use of talcum powder contaminated with asbestos).^3,4,25,26^ Asbestos is banned in many countries around the world, so an assessment of mesothelioma in India requires special focus, as large quantities of asbestos are imported to make asbestos roofing (among other products) and also exported to other low-income countries.^27^ Asbestos is also present in marble quarries and mines, and it is possible that workers may be at an additional risk of asbestosis and mesothelioma, apart from the silicosis risk, as they may be unaware of their exposure to asbestos.^28^ A limitation of our study is that although asbestos exposure has been shown to be the major cause of mesothelioma, there may be other causative agents, such as fibres due to sugarcane farming (although a positive correlation has not been found in some studies),^29,30^ and carbon nanotubes.^31^ Another potential limitation is that the duplication of recorded cases has not been accounted for, as has been suggested for cancer record-keeping.^32^ As such, a patient who has visited multiple hospitals may have had multiple entries in this study. This is something that the NCDIR database takes into consideration, but it was not possible to assess in the context of this study.

This study examines whether mesothelioma is rare in India. The results presented do not support this – instead, they suggest the opposite. The level of underreporting should serve as an alarm bell for this so called ‘rare malignancy’: between 2012 and 2022 (and 2023 in some hospitals), we found 2,213 cases of mesothelioma. The study also highlights the large number of hospitals that remain outside of the Indian National Cancer Registry Programme.

## CONCLUSION

The major conclusion of our study is that a large number of mesothelioma cases are unreported. This means that past asbestos exposure in patients with mesothelioma is unrecognised, which is alarming. We believe this demonstrates the need to better regulate asbestos use or, better yet, ban it entirely as many countries around the world have done. If this preventable disease creates a burden on the healthcare system, it may cost more than the benefits of asbestos use, when safer substitutes are available. It is also recommended that cancer record-keeping be improved in India. This can be done by 1) making cancer notifiable across the country with a PBCR in every State; 2) ensuring that data collection includes common identity mechanisms so that an individual patient’s data can be easily recognised; 3) making the cancer registry programme mandatory instead of voluntary; and 4) recognising and replicating the work done by states such as Tamil Nadu, which has created a state-based cancer registry, across all the States and Union Territories of India.

## Supplementary Material


