# Supplementary material for: Analysis of mesothelioma cases and National Cancer Registry data to assess asbestos exposure in India
Source: Public Health Action. 2024 Mar 1;14(1):30–3. doi: 10.5588/pha.24.0003 (PMC11122712; doi:10.5588/pha.24.0003)
Supplement: Supplementary file 1 [file iutld_pha_24.0003_supplementarydata1.pdf]

http://dx.doi.org/10.5588/pha.24.0003

SUPPLEMENTARY DATA

Analysis of mesothelioma cases and National Cancer Registry data to assess asbestos exposure in India

Supplementary Table S1

1	Homi Bhabha Cancer Hospital & Research Centre, Visakhapatnam	**Andhra Pradesh**			1	0	1	0	1	0	3	2	1	0	**9**
2	Sri Venkateswara Institute of Medical Sciences, Tirupati	**Andhra Pradesh**	1	0	2	1	1	2	1	0	2	1	1		**12**
3	Govt. Hospital for Chest and Communicable Diseases, Guntur	**Andhra Pradesh**	0	0	0	0	0	0	0	0	0	0	0		**0**
4	Dr Bhubaneshwar Barooah Cancer Institute Guwahati	**Assam**	0	1	2	2	2	2	0	0	0	0	0		**9**
5	Patna Medical College Hospital, Patna	**Bihar**	0	1	0	0	0	0	0	0	0	0	0	0	**1**
6	Homi Bhaba Cancer Hospital and Research Centre, Muzaffarpur	**Bihar**											0	0	**0**
7	All India Institute of Medical Sciences, Raipur	**Chhattisgarh**						6							**6**
8	Dr Bhimrao Ambedkar Memorial Hospital, Raipur	**Chhattisgarh**	145	195	229	47	83	57	109	3	84	50	59		**1061**
9	Goa Medical College	**Goa**	0	0	0	0	0	0	2	1	0	2	0	1	**6**

10	Guru Gobind Singh Hospital, Jamnagar	**Gujarat**	1	1	0	0	0	0	2	0	0	1	0	0	**5**
11	Sir Sayajirao General Hospital, Vadodara	**Gujarat**		1	2	2	1	2	1	1	1	1	1		**13**
12	Gujarat Cancer and Research Centre, Ahmedabad	**Gujarat**	13	24	28	32	27	28	27	32	19	35	24	14	**303**
13	GMERS Medical College and Hospital Navsari	**Gujarat**											0		**0**
14	Sardar Vallabhai Patel Institute of Medical and Research, Ahmedabad	**Gujarat**	2												**2**
15	Sir Takht Sinhji Hospital, Bhavnagar	**Gujarat**	0	1	0	1	0	0	0	0	0	1	0		**3**
16	National Cancer Institute, AIIMS, Jhajjar	**Haryana**						7							**7**
17	Pandit Bhagwat Dayal Sharma Post Graduate Institute of Medical Sciences, Rohtak	**Haryana**	1	0	0	0	1	0	0	0	0	0	0	0	**2**
18	Dr Rajendra Prasad Government Medical College, Tanda	**Himachal Pradesh**								1	0	0	1	0	**2**
19	Indira Gandhi Medical College, Shimla	**Himachal Pradesh**	0	0	0	0	0	0	0	0	0	0	1	0	**1**
20	Bokaro Steel Plant Hospital	**Jharkhand**	0	0	0	1	0	0	0	0	0	0	0	0	**1**
21	Karnataka Institute of Medical Sciences, Hubli	**Karnataka**	1	1	3	0	0	1	0	0	0	0	4	0	**10**
22	Mysore Medical College and Research Institute and KR Hospital, Mysore	**Karnataka**	0												**0**
23	Kidwai Memorial Institute of Oncology, Bengaluru	**Karnataka**	0	0	0	1	0	0	0	0	0	0	0	0	**1**
24	Malabar Cancer Centre	**Kerala**	2	1	2	3	2	1	1	1	1	2			**16**
25	T.D. Medical College Hospital Alappuzha	**Kerala**				4									**4**
26	General Hospital Ernakulum	**Kerala**	0	0	0	0	0	0	1	3	1	0	0	0	**5**

27	Govt. Medical College Hospital, Kannur	**Kerala**	0	1	0	0	0	0	0	0	0	0	0	0	**1**
28	Govt. Medical College Hospital, Kollam	**Kerala**						0	0	0	0	0	0	0	**0**
29	Govt. Medical College Hospital, Kottayam	**Kerala**	1	2	3	5	4	2	4	6	3	0			**30**
30	Bhopal Memorial Hospital and Research Centre	**Madhya Pradesh**	2												**2**
31	AIIMS Bhopal	**Madhya Pradesh**						7							**7**
32	State Cancer Institute, Netaji Subhash Chandra Bose Medical College Jabalpur	**Madhya Pradesh**	0	0	0	0	0	0	0	0	0	0	0	0	**0**
33	Shyam Shah Medical College, Rewa	**Madhya Pradesh**				0	0	0	0	0	1	2	1	0	**4**
34	Tata Memorial Hospital	**Maharashtra**	18	16	19	6	20	40	30	47	2	11	42	11	**262**
35	ESIC, ODC, Mumbai	**Maharashtra**	0	0	0	0	0	0	0	0	0	0	0		**0**
36	Department of Radiation Oncology Cama & Albless Hospital Mumbai	**Maharashtra**	0	0	0	0	0	0	0	0	0	0	0	0	**0**
37	Bhaba Atomic Research Centre (Hospital)	**Maharashtra**	1	0	0	0	0	0	0	0	0	0		0	**1**
38	North Eastern Indira Gandhi Regional Institute of Health and Medical Sciences Shilong	**Meghalaya**	0	0	0	0	0	0	2	0	0	1	0		**3**
39	Rajinder Hospital, Patiala	**Punjab**	0	0	0	0	0	0	0	0	0	0	0	0	**0**
40	Homi Bhabha Cancer Hospital, Sangrur	**Punjab**						0	0	1	1	0	0	0	**2**
41	Homi Bhabha Cancer Hospital and Research Centre, Mullanpur	**Punjab**											0	0	**0**
42	Sawai Man Singh Hospital , Jaipur	**Rajasthan**	0	0	0	0	0	0	0	0	0	0	0		**0**
43	Dr Sampurna Nand Medical College and Mathuradas Mathur Hospital, Jodhpur	**Rajasthan**		0	0	0	0	0	0	0	0	0	1	1	**2**

44	Govt Medical Hospital, Kota	**Rajasthan**	0	0	0	0	0	0	0	0	0	0	0	0	**0**
45	Acharya Tulsi Cancer Hospital and Research Institute	**Rajasthan**	0	1	2	6	1	3	3	5	3	5	4	5	**38**
46	TNCRP- Govt. Rajiv Gandhi General Hospital, Chennai	**Tamil Nadu**	25	8	24	27	28	18	29	19	8	18	13	4	**221**
47	TNCRP- Government Royapettah Hospital, Chennai														
48	TNCRP- Government Stanley Medical College, Chennai														
49	TNCRP- Institute of Obstetrics and Gynaecology, Chennai														
50	TNCRP- Madras Cancer Care Foundation, Chennai (Kumaran Hospital)														
51	TNCRP- The Tamil Nadu Government Multi Super Speciality Hospital, Chennai														
52	TNCRP- Apollo Cancer Centre														
53	TNCRP- Sri Ramachandra Medical College and Hospital														
54	TNCRP- Govt. Arignar Anna Memorial Cancer Hospital & Research Institute, Kanchipuram														
55	TNCRP- Govt. Viluppuram Medical College and Hospital														
56	TNCRP- Kovai Medical Centre and Hospital, Coimbatore														
57	TNCRP- G. Kuppuswamy Naidu Memorial Hospital, Coimbatore														
58	TNCRP- Erode Cancer Centre, Thindal, Erode														

59	TNCRP- Thanjavur Cancer Centre, Thanjavur														
60	TNCRP- Govt Tirunelveli Medical College and Hospital, Tirunelveli														
61	TNCRP- International Cancer Centre, Neyyoor														
62	TNCRP- Jawaharlal Institute of Postgraduate Medical Education and Research, Regional Cancer Centre, Puducherry														
63	Command Hospital Chennai	**Tamil Nadu**				1	0	0	1	2	0	4	1	0	**9**
64	Coimbatore Medical College Hospital	**Tamil Nadu**	2	0	0	1	2	1	2	0	0	0	0	1	**9**
65	Kalyan Singh Super Speciality Cancer Institute Lucknow	**Uttar Pradesh**										0	0		**0**
66	Institute of Medical Sciences, Banaras Hindu University, Varanasi	**Uttar Pradesh**	0	0	0	0	0	0	0	0	0	0	0	0	**0**
67	Mahamana Pandit Madan Mohan Malviya Cancer Centre, Varanasi	**Uttar Pradesh**							3	9	7	9	7	7	**42**
68	Jawaharlal Medical College, AMU, Alighar	**Uttar Pradesh**							0	0	0	0	0		**0**
69	Lala Lajpat Rai Memorial College, Meerut	**Uttar Pradesh**	0	0	0	0	0	0	0	0	0	0	0	0	
70	Chittaranjan National cancer institute	**West Bengal**					2	4	2	4	0	1			**13**
71	Command Hospital	**West Bengal**	0	0	0	0	0	0	0	0	0	0	0		**0**
72	ESIC Hospital, Joka	**West Bengal**				1	0	0	1	2	0	4	1	0	**9**
73	Government Medical College and Hospital	**Chandigarh**						1							**1**
74	Nehru Hospital Postgraduate Institute of Medical Education and Research	**Chandigarh**			0	2	0	0	0	1	0	0	0		**3**

75	Lady Hardinge Medical College & Srimati Suchetra Kriplani Hospital	**Delhi**			0	0	0	0	0	0		0	0	0	**0**
76	Vardhaman Mahaveer Medical College and Safdarjung Hospital	**Delhi-UT**	2	0	0	0	0	0	3	0	0	1			**6**
77	Delhi State Cancer Institutes	**Delhi-UT**	5	5	3	4	4	1	5	1	0	0	0		**28**
78	Kalavati Saran Childrens Hospital	**Delhi-UT**	0	0	0	0	0	0	0	0	0	0	0		**0**
79	Bhim Rao Ambedkar Rotary Cancer Centre, AIIMS Delhi	**Delhi-UT**	32												**32**
80	National Institute of Tuberculosis and Lung Diseases	**Delhi-UT**													**0**
81	Lok Nayak Hospital	**Delhi-UT**	0	0	0	0	0	0	0	0	0	0	0	0	
82	Government Medical College, Jammu	**J&K-UT**			1	0	1	1	0	1	1	0	0	1	**6**
83	Govt Medical College, Karanagar, Srinagar	**J&K-UT**			0	0	1	0	0	0	0	0	1	1	**3**
															**2213**

Table S1: The list of hospitals state-wise of which data has been included in the study.

AIIMS = All India Institute of Medical Sciences; AMU = Aligarh Medical University; GMERS = Gujarat Medical & Education Research Society; Govt.: Government; ESIC= Employee State Insurance Corporation; ODC = Occupational Disease Centre; PGIMS = Post Graduate Institute of Medical Sciences; TNCRP = Tamil Nadu Cancer Registry; UT = Union Territory

Note 1: For the missing years, the hospital did not record data, or the hospital did not function or did not have a department dealing with diagnosis/treatment of cancer.

Note 2: Data from hospital number 8 seemed more than the average, but since it was obtained through the same methodology, i.e., the Right to

Information Act 2005, duly certified by the hospital, it has been included as it is.

*Date for the year 2023 is up till the data available and not for the full year as the data was collected before the end of 2023.
